# The Genotoxic Stress Sensor ZBP1 Drives Tau Pathology

**DOI:** 10.3390/cells15070591

**Published:** 2026-03-26

**Authors:** Jessica M. Thanos, Olivia C. Campbell, Nick R. Natale, Ana Royo Marco, Michael A. Puchalski, John R. Lukens

**Affiliations:** 1 Center for Brain Immunology and Glia (BIG), Harrison Family Translational Research Center in Alzheimer’s and Neurodegenerative Diseases, Department of Neuroscience, University of Virginia, Charlottesville, VA 22908, USA; rpe2je@virginia.edu (O.C.C.); nrn2k@virginia.edu (N.R.N.); ar5wz@virginia.edu (A.R.M.); map3fz@virginia.edu (M.A.P.); 2 Neuroscience Graduate Program, University of Virginia, Charlottesville, VA 22908, USA; 3 Global Biothreats Graduate Training Program, University of Virginia, Charlottesville, VA 22908, USA

**Keywords:** Alzheimer’s disease, tauopathy, neuroinflammation, ZBP1, genotoxic stress, neurodegenerative disease, nucleic acid sensing, innate immunology

## Abstract

**Highlights:**

**What are the main findings?**

- Genetic ablation of the innate immune genotoxic stress sensor ZBP1 protects against tauopathy and associated neuronal loss.
- Knockdown of ZBP1 in tauopathy dampens neuroinflammation, limits gliosis, and suppresses the expression of necroptosis- and IFN-related genes.

**What are the implications of the main findings?**

- These findings provide a proof of principle that ZBP1 can be targeted to beneficially modulate the major neuropathological hallmarks of tauopathy, including gliosis, neuroinflammation, and neuronal loss.
- These results suggest that inhibiting ZBP1 may be an effective therapeutic strategy to slow or treat primary or secondary tauopathies, such as frontotemporal dementia and late stages of Alzheimer’s disease, as well as related neurodegenerative conditions.

**Abstract:**

Genotoxic stress, which includes DNA damage and the mis-localization of DNA and RNA, is a defining feature of tauopathies, Alzheimer’s disease, and several other neurodegenerative disorders. Recent findings indicate that activation of the innate immune system in response to genotoxic stress can drive harmful neuroinflammation, compromise neuronal integrity, and promote neurodegeneration. Multiple innate immune sensors of genotoxic stress have recently been discovered, but the contributions of many of these emerging nucleic acid–sensing pathways in neurodegenerative disease pathogenesis remain largely unexplored. Z-DNA binding protein 1 (ZBP1) is one such recently discovered genotoxic stress sensor that has been shown to incite various forms of cell death as well as proinflammatory cytokine production in response to left-handed Z conformations of DNA (Z-DNA) and RNA (Z-RNA). Here, we show that ZBP1 deletion provides protection against tau pathology and neuronal loss in the PS19 mouse model of tauopathy. Moreover, we find that this rescue of tauopathy seen with ZBP1 ablation is associated with dampened activation of microglia and astrocytes. These findings identify ZBP1 as a pivotal genotoxic stress sensor that drives tau pathology, gliosis, and neuronal loss in tauopathy. This work further suggests that targeting ZBP1 may offer a therapeutic strategy to treat tau-mediated neurodegenerative disease.

## 1. Introduction

Tauopathies are a diverse group of neurodegenerative diseases that include frontotemporal dementia, Alzheimer’s disease (AD), progressive supranuclear palsy, and others. Tauopathy disease progression is triggered by the accumulation of phosphorylated-tau (p-tau), which forms the backbone of the neurofibrillary tangles that propagate neuronal dysfunction and death. Recent studies indicate that unchecked neuroinflammation is a major driver of p-tau formation. More specifically, proinflammatory cytokine production has been shown to trigger excessive activation of stress kinases that phosphorylate tau in neurons [1,2,3,4,5,6]. While mounting evidence clearly implicates neuroinflammation in driving tauopathy, the specific molecular regulators of the dysregulated immune responses underlying tauopathy progression are just beginning to be uncovered.

Genotoxic stress in the form of DNA damage and DNA/RNA mis-localization is a hallmark of tauopathies, AD, and various other neurodegenerative diseases [7,8,9]. In the context of AD, recent studies have shown that activation of the innate immune system in response to genotoxic stress can trigger detrimental neuroinflammation, disrupt neuronal health, and lead to neurodegeneration [2,10,11,12]. Most notably, mounting evidence has revealed that detection of displaced DNA in the cytosol by the innate immune nucleic acid sensor cGAS-STING leads to heightened proinflammatory cytokine production, exacerbated neuropathology, and accelerated cognitive decline in various mouse models of AD [2,10,11,12]. Notably, blockade of cGAS-STING was shown to ameliorate neurodegenerative disease progression in models of both AD-associated amyloidosis and tauopathy [2,10,11,12], which suggests that targeting the innate immune response to genotoxic stress could offer a strategy to treat AD. In addition to cGAS-STING, multiple other innate immune-based genotoxic stress sensors have recently been discovered [13,14]. However, the roles of many of these emerging innate immune nucleic acid-sensing pathways in AD and other tauopathies have not been studied in great detail to date.

One such recently identified genotoxic stress sensor is Z-DNA-binding protein 1 (ZBP1; also previously referred to as DNA-dependent activator of IFN-regulatory factors or DAI). ZBP1 is a cytosolic innate immune sensor that detects double-stranded left-handed Z conformations of DNA (Z-DNA) and RNA (Z-RNA) [15,16]. While the majority of DNA and RNA exist in right-handed B-conformations, DNA and RNA can undergo conformational changes and acquire left-handed Z-conformations under conditions of genomic, metabolic, and oxidative stress [17]. ZBP1 has been most extensively studied in the context of viral infection, where it has been shown to be centrally involved in coordinating NF-κB signaling, proinflammatory cytokine production, and various discrete forms of cell death [18,19,20,21,22]. In addition to its prominent involvement in mounting protective antiviral immunity, studies conducted over the last few years have also begun to uncover pivotal roles for ZBP1 in driving autoinflammatory responses to host-derived Z-DNA/Z-RNA [23,24,25,26,27,28,29,30]. Recent studies have shown that Z-DNA/Z-RNA levels are elevated in the brains of AD patients and further indicate that this is accompanied by heightened ZBP1 activation and related downstream signaling [31,32,33]. Emerging evidence also suggests that ZBP1 is an instigator of neuroinflammation and brain pathology in models of AD-associated amyloidosis [31,34]. However, the role of ZBP1 in tauopathies remains largely unexplored.

Here, we investigated how ZBP1 shapes tau-mediated neurodegenerative disease progression in the Tau P301S (PS19) mouse model of tauopathy. By crossing PS19 mice to *Zbp1*-deficient mice, we show that loss of ZBP1 function markedly reduces pathogenic tau species, including multiple phospho-epitopes and misfolded/conformational tau. ZBP1 deletion also dampens microglial activation and astrocyte reactivity in the brains of PS19 mice. Single-nucleus RNA sequencing (snRNA-seq) of the hippocampus reveals that ZBP1 ablation blunts disease-associated transcriptional programs in microglia and astrocytes. Furthermore, genetic ablation of ZBP1 in tauopathy protects against neuronal loss and shifts excitatory neuron transcriptional profiles away from stress responses toward synaptic function and plasticity gene expression modules. Taken together, these data identify ZBP1 as a central innate immune mediator that links tau pathology to maladaptive glial activation and neuronal loss. Moreover, these findings suggest that targeting ZBP1-dependent signaling may offer a strategy to limit tau-driven neurodegeneration.

## 2. Methods

### 2.1. Animal Care and Maintenance

All mouse experiments were performed in accordance with the relevant guidelines and regulations of the University of Virginia and approved by the University of Virginia Animal Care and Use Committee. Tau P301S (PS19; The Jackson Laboratory, Bar Harbor, ME, USA; stock no. 024841) [35] and Zbp1-KO (Cyagen Bioscience, Santa Clara, CA, USA; stock no. S-KO-16146) mice were obtained and crossed to generate PS19^+/−^ *Zbp1*^+/+^ (denoted as PS19) and PS19^+/−^ *Zbp1*^−/−^ (denoted as PZKO) experimental mice. Mice were housed under specific pathogen-free conditions with controlled temperature (21 ± 1.5 °C) and humidity (50 ± 10%), maintained on a standard 12-h light/dark cycle and provided food and water ad libitum. Male and female mice were used in all studies in similar proportions, regardless of genotype, unless otherwise specified.

### 2.2. Tissue Collection and Cryopreservation

Mice were exposed to CO_2_ to induce deep anesthesia, then transcardially perfused with 20 mL of 1× Dulbecco’s phosphate-buffered saline (PBS; Thermo Fisher Scientific, Waltham, MA, USA; cat. no. 14190144) and decapitated as a secondary method of euthanasia. Brains were collected and bisected along the sagittal plane to yield two hemispheres, one for ELISA or sequencing experiments, and the other for immunofluorescence studies. For ELISA and sequencing experiments, hippocampal tissue was micro-dissected from one hemisphere, snap-frozen on dry ice, and stored at −80 °C until further use. For immunofluorescence studies, remaining hemispheres were immersion-fixed in 15 mL of 4% paraformaldehyde (PFA; Electron Microscopy Sciences, Hatfield, PA, USA; cat. no. 15714-S) in PBS overnight at 4 °C, washed in PBS three times at room temperature (RT), and transferred to 30% sucrose in PBS at 4 °C until they had fully sunk. Hemibrains were then washed again in PBS, embedded in optimal cutting temperature (OCT; Sakura Finetek USA, Torrance, CA, USA; cat. no. 4583) compound, snap-frozen on dry ice, and stored at −80 °C prior to sectioning. Cryopreserved hemibrains were sectioned coronally at 40 µm thickness using a cryostat (Leica Biosystems, Nussloch, Germany; cat. no. CM1950) to recover the entire hippocampal region and stored in 0.05% sodium azide (NaN_3_; Ricca Chemical, Arlington, TX, USA; cat. no. 71448-16) in PBS at 4 °C to minimize microbial growth prior to staining.

### 2.3. Enzyme-Linked Immunosorbent Assay (ELISA)

Frozen micro-dissected hippocampi were thawed at room temperature (RT) and mechanically homogenized in 250 µL tissue protein extraction reagent (T-PER; Thermo Fisher Scientific; cat. no. 78510) supplemented with 1× PhosSTOP phosphatase inhibitor (PhosSTOP; Roche, Basel, Switzerland; cat. no. C764L25) and 1× cOmplete protease inhibitor cocktails (Roche; cat. no. 11-697-498-001). Homogenates were centrifuged at 16,000× *g* for 10 min at 4 °C to recover soluble proteins in the supernatant. To extract insoluble proteins, remaining hippocampal pellets (approximately 25 µL) were resuspended in 150 µL 5 M guanidine hydrochloride (GuHCl; Thermo Fisher Scientific; cat. no. 24115) in 50 mM Tris base (pH 8.0) and incubated for 3 h at RT on a rocker at maximum speed. Samples were diluted 1:8 in PBS containing PhosSTOP and cOmplete inhibitors and centrifuged at 16,000× *g* for 20 min at 4 °C to recover insoluble proteins in the supernatant. Total soluble and insoluble protein concentrations were quantified against bovine serum albumin (BSA; Thermo Fisher Scientific; cat. no. BP1600-100) standards using the Protein 660 reagent (Thermo Fisher Scientific; cat. no. 22660) according to the manufacturer’s instructions and stored at −80 °C until use. Soluble and insoluble protein lysates were diluted in 1× PBS at 1:10 and 1:1000, respectively. Phosphorylated T181 and S396 tau levels were measured by sandwich ELISA according to the manufacturer’s instructions. Briefly, samples and standards were added to coated strips and incubated with detector antibody solution. Strips were washed in wash buffer, incubated with IgG–HRP solution, washed again, and incubated with stabilized chromogen. Reactions were stopped with stop solution, and absorbance was read at 450 nm on a plate reader. Blank values were subtracted from optical density readings and normalized to total protein. Normalized values were reported as picograms of target protein per milligram (pg/mg) of total protein.

### 2.4. Immunofluorescence

All immunofluorescence incubations were performed under gentle agitation on a laboratory rocker. Free-floating sections were blocked and permeabilized in 2% donkey serum, 1% BSA, 0.1% Triton X-100, and 0.05% Tween 20 in PBS (immunofluorescence [IF] buffer) for 2 h at RT. For staining with mouse-derived tau antibodies, 2 drops of mouse-on-mouse blocking reagent (Vector Laboratories, Newark, CA, USA; cat. no. MKB-2213-1) were added per 2.5 mL of IF buffer according to the manufacturer’s instructions. Sections were incubated with primary antibodies diluted in IF buffer overnight at 4 °C, washed three times in 0.05% Tween-20 in PBS (wash buffer) for 10 min at RT, and incubated with secondary antibodies in IF buffer for 2 h at RT. Sections were washed again, counterstained with 20 µg/mL DAPI (Sigma-Aldrich, St. Louis, MO, USA; cat. no. D9542) in PBS for 10 min at RT, and returned to PBS. Stained sections were mounted in ProLong Gold antifade reagent (Invitrogen, Carlsbad, CA, USA; cat. no. P36930) onto charged glass microscope slides (Thermo Fisher Scientific; cat. no. 1255015), coverslipped (Thermo Fisher Scientific; cat. no. 12-544C), cured overnight at RT, and stored at 4 °C protected from light until imaging. The following primary antibodies/fluorescent stains were used: AT8 (Thermo Scientific #MN1020; 1:500), MC1 (Peter Davies, Albany, NY, USA; 1:500), anti-IBA1 (Synaptic Systems, Goettingen, Germany; cat. no. 234-308; 1:1000), anti-CD68 (Bio-Rad, Hercules, CA, USA; cat. no. MCA1957; 1:1000), anti-P2RY12 (Abcam, Cambridge, UK; cat. no. ab300140; 1:1000), anti-GFAP (Thermo Scientific cat. no. 13-0300; Waltham, MA, USA), NeuroTrace 500/525 (Nissl; Thermo Scientific cat. no. N21480; 1:200), and anti-MAP2 (MilliporeSigma, Burlington, MA, USA; cat. no. AB5622; 1:200). All secondary antibodies were raised in donkey and conjugated to Alexa Fluor fluorophores (Invitrogen; 1:1000).

### 2.5. Image Acquisition and Analysis

All images were acquired on a Stellaris 5 confocal microscope using the LAS X software (v4.8.29271; Leica Microsystems, Mannheim, Germany) at 8-bit depth, 512 × 512-pixel resolution, and 1× digital zoom. All images encompassed entire sections to ensure consistent representation of bright-edge artifacts across samples. For whole-hippocampal regions of interest (HP ROI), tile scans were acquired at 10× objective magnification with 0.5-µm z-steps. A 10% overlap was set to enable accurate stitching, and tiles were stitched using the “None” option in LAS X to avoid additional blending or intensity correction. For fields of view (FOV) within hippocampal subregions (CA1, CA2, CA3, or DG), images were acquired at 40× magnification with 0.5-µm z-steps. All image processing and 2-dimensional (2D) analyses were performed in Fiji (ImageJ v1.54p; NIH, Bethesda, MD, USA). The Bio-Formats plugin was used to convert Leica image files (LIF) to TIFF format and to generate individual Z-stacks for each tile or image, split by channel. Z-stacks for each channel were converted to maximum-intensity projections, and automatic thresholding methods were selected for each channel to maximize signal-to-noise and account for background variability. Consistent thresholding parameters were applied to all images for each stain, regardless of animal, sex, or genotype. The “Measure” function was then used to obtain %area values, which were reported as coverage per FOV or ROI. All steps were implemented using custom Fiji macros to ensure consistent, batch-wise processing across samples.

### 2.6. Single-Nucleus RNA Sequencing (snRNA-Seq)

Snap-frozen hippocampi were collected from 9-month-old male PS19 and PZKO mice and submitted to GENEWIZ (Azenta Life Sciences, Burlington, MA, USA) for nuclei isolation, library preparation, and RNA sequencing. For each genotype, hippocampi from individual animals were processed separately. Nuclei were isolated using a proprietary detergent-based lysis buffer and gentle mechanical disruption, filtered to remove debris, and counted by fluorescent dye exclusion. Single-nucleus 3′ RNA libraries were generated using the Chromium Single Cell 3′ Reagent Kit (10× Genomics, Pleasanton, CA, USA) according to the manufacturer’s protocol and loaded for target capture of ~6000 nuclei per sample. Libraries were quality-checked by capillary electrophoresis and fluorometric quantification and sequenced on an Illumina NovaSeq platform. Raw BCL files were converted to FASTQ and processed with Cell Ranger (v7.0.1; 10× Genomics) using the count pipeline and the mm10 mouse reference genome to generate gene-by-cell count matrices for each sample. Cell Ranger outputs were then imported into R and used to construct Seurat objects. Ambient RNA contamination was estimated and corrected with SoupX, and low-quality nuclei were removed using the following thresholds: <600 UMIs, <300 detected genes, or >5% mitochondrial transcripts. Putative doublets were identified and removed using DoubletFinder, and high-quality singlets were then normalized, log-transformed, scaled, and used to identify highly variable genes. The PS19 and PZKO datasets were integrated using the harmony workflow in Seurat, followed by graph-based clustering with UMAP. Cluster-defining markers were identified and used to assign major cell-type identities (e.g., excitatory neurons, inhibitory neurons, microglia, astrocytes, oligodendrocytes, and endothelial cells). For each cell type containing >50 nuclei per genotype, differential expression analysis was performed using DESeq2 on pseudobulked counts. Volcano plots and feature plots were generated using Seurat and EnhancedVolcano. Significant and differentially expressed genes (DEGs) were used as inputs for functional and pathway enrichment analyses in EnrichR. Significantly enriched terms were ranked in ascending order by adjusted *p*-value, with the topmost (i.e., most significantly enriched) terms shown as bar plots.

### 2.7. Statistical Testing

For immunofluorescence (IF) and ELISA experiments, data are representative of two independent experiments, and male and female mice were used in similar proportions across genotypes. Each IF experiment included a minimum of 5 mice per genotype, with 2–3 sections per mouse and 1 image analyzed per section. ELISA experiments included one technical measurement per mouse sample. All IF and ELISA visualizations were generated in Prism (v11.0.0; GraphPad, San Diego, CA, USA). Coverage data from IF studies were analyzed using linear mixed-effects models implemented in the lme4 package in R, with mouse treated as a random effect to account for multiple sections per animal. ELISA data were analyzed using unpaired Student’s *t* tests in Prism (GraphPad). For single-nucleus RNA-sequencing studies, only male mice were used, and each sequenced sample consisted of pooled hippocampi from 5 males per genotype.

## 3. Results

### 3.1. Loss of ZBP1 Protects Against Tauopathy

**Figure 1:**
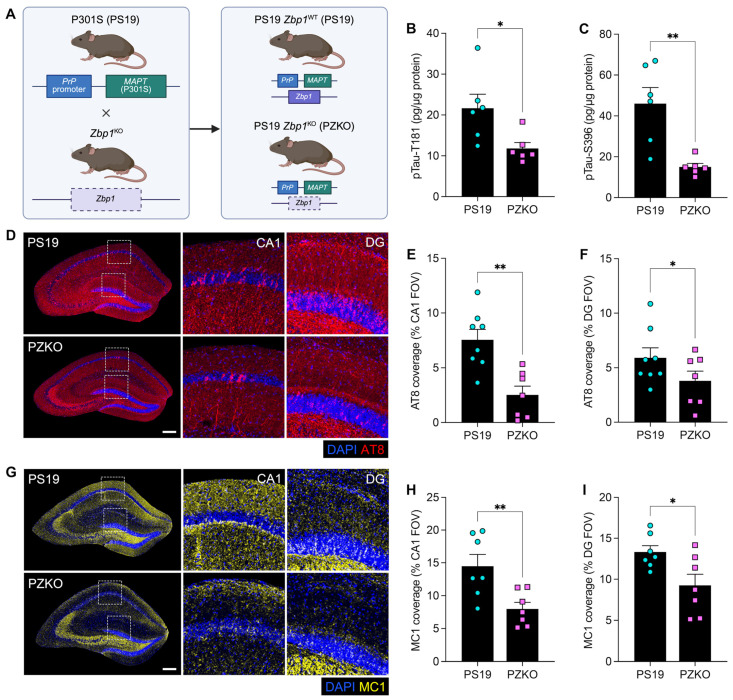
ZBP1 ablation limits pathogenic tau burden. (**A**) Schematic of the breeding strategy used to study the effects of ZBP1 deletion on tauopathy pathogenesis. P301S (PS19) and ZBP1-null (*Zbp1*^KO^) mice were crossed to generate PS19 *Zbp1*^KO^ (abbreviated as PZKO) mice and PS19 *Zbp1*^WT^ (abbreviated as PS19) littermate controls. Brains were harvested from PZKO and PS19 mice at 9 months of age to evaluate tau pathology by ELISA and immunofluorescence staining. (**B**) T-PER soluble p-tau T181 as picograms per milligrams (pg/mg) of total protein. (**C**) Guanidine-HCl soluble p-tau S396 as pg/mg of total protein. (**D**) Representative immunofluorescence (IF) images of hippocampal DAPI (blue) and AT8 (pTau-S202/T205; red) staining (scale bar = 200 µm; insets depict enlarged views of CA1 and DG within dashed frames). (**E**) AT8^+^ area as a percentage of the total field of view (FOV) within CA1. (**F**) AT8^+^ area as a percentage of the total FOV within DG. (**G**) Representative IF images of hippocampal DAPI (blue) and MC1 (misfolded/conformational tau; yellow) staining (scale bar = 200 µm; insets depict enlarged views of CA1 and DG within dashed frames). (**H**) MC1^+^ area as a percentage of the total FOV within CA1. (**I**) MC1^+^ area as a percentage of the total FOV within DG. Each data point represents an individual mouse and the error bars represent mean ± SEM. Statistical significance for ELISA experiments (**B**,**C**) was determined by unpaired Student’s *t*-test. Linear mixed-effects regression (LMER) testing was used for IF experiments (**E**,**F**,**H**,**I**). * *p* < 0.05, ** *p* < 0.01.

To explore a role for ZBP1 in tauopathy pathogenesis, we crossed ZBP1-deficient mice (*Zbp1*^KO^; Figure 1A) onto the PS19 mouse model of tauopathy [35]. The resulting PS19 *Zbp1*^KO^ (PZKO) mice and their PS19 *Zbp1*^WT^ (PS19) littermates are compared throughout this study. In humans with tauopathies and in PS19 mice, specific epitopes of tau phosphorylation and conformational misfolding enhance pathogenicity and also serve as reliable diagnostic markers of disease progression. To evaluate whether ZBP1 deletion alters hippocampal accumulation of pathogenic tau species, we first quantified established markers of p-tau and tau misfolding. Enzyme-linked immunosorbent assays (ELISAs) of whole-hippocampal lysates showed reduced levels of soluble p-tau T181 (Figure 1B) and insoluble p-tau S396 (Figure 1C) in the PZKO hippocampus compared with PS19 littermates. Consistently, we found that genetic ablation of ZBP1 in PS19 mice markedly reduced levels of p-tau in the cornu ammonis 1 (CA1) and dentate gyrus (DG) of the hippocampus at 9 months of age in comparison to PS19 littermate controls (Figure 1D–F). Next, we performed immunofluorescence staining with the MC1 antibody, which detects pathogenic conformations of tau that have been linked with AD progression [36]. In line with our AT8 p-tau staining findings (Figure 1D–F), we also observed significantly reduced MC1 staining throughout the hippocampus of ZBP1-deficient PS19 mice, relative to PS19 littermate controls (Figure 1G–I). Taken together, these findings indicate that deleting ZBP1 represses tau pathology in PS19 mice.

### 3.2. ZBP1 Deletion Restricts Disease-Associated Microglial States in Tauopathy

**Figure 2:**
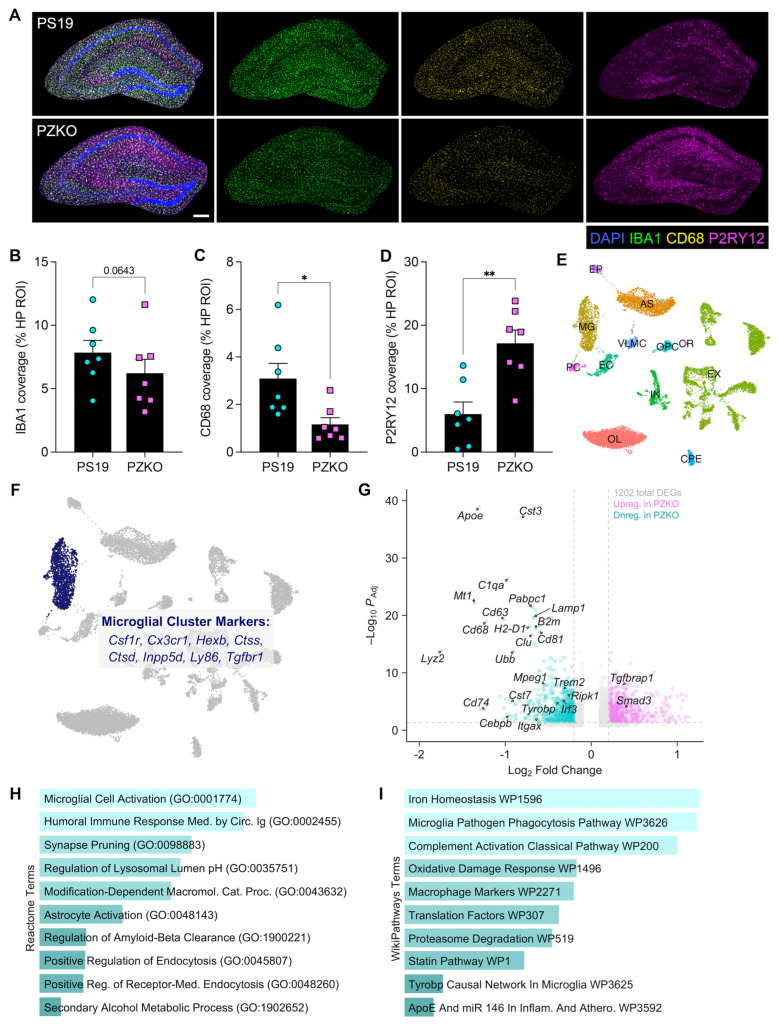
Tauopathy-driven microglial activation is dampened with ZBP1 deletion. (**A**) Representative IF images of hippocampal DAPI (blue), IBA1 (green), CD68 (yellow), and P2RY12 (magenta) staining (scale bar = 200 µm). (**B**) IBA1^+^ area as a percentage of the total hippocampal region of interest (HP ROI). (**C**) CD68^+^ area as a percentage of total HP ROI. (**D**) P2RY12^+^ area as a percentage of total HP ROI. (**E**) Uniform Manifold Approximation and Projection (UMAP) plot depicting cell clusters identified by single-nucleus RNA sequencing (snRNA-seq) of hippocampal tissue pooled from PS19 and PZKO mice at 9 months of age. Downstream analysis was performed using the Seurat package following standard preprocessing, normalization, dimensionality reduction, and clustering pipelines. (Abbreviations: AS = astrocyte; OL = oligodendrocyte; MG = microglia; OPC = oligodendrocyte precursor cells; EX = excitatory neuron; EC = endothelial cell; IN = inhibitory neuron; PC = pericyte; VLMC = vascular leptomeningeal cell; CP = choroid plexus [epithelial cell]; EP = ependymal [epithelial cell]). (**F**) UMAP plot with highlighted microglial cluster (labeled ‘MG’) and canonical identity markers curated from significantly enriched genes identified by ‘FindAllMarkers’ function in Seurat (v5.2.0). (**G**) Volcano plot depicting differentially expressed genes (DEGs) in the PZKO microglial cluster compared to that of the PS19 group. The combined microglia cluster was subsetted and genotype comparisons were tested using the MAST algorithm in Seurat. DEGs with –Log_2_ Fold Change (avg_log2FC) ± 0.25 and an adjusted *p*-value less than 0.05 were plotted using the EnhancedVolcano package. Genes are colored based on directionality of change (magenta: upregulated in PZKO; teal: downregulated in PZKO) and genes of interest are labeled on the plot. (**H**) Reactome Pathway Database terms enriched in downregulated DEGs in PZKO microglia. DEGs used in volcano plots were used as inputs to the EnrichR website portal for pathway enrichment analysis. Plot shows the top significantly enriched terms, ranked by ascending adjusted *p*-value. (**I**) WikiPathway Database terms enriched in downregulated DEGs in PZKO microglia using the same criteria and methods as for the Reactome Pathway Database. Plot shows the top significantly enriched terms, ranked by ascending adjusted *p*-value. Statistical significance between experimental groups was calculated by linear mixed effects modeling. * *p* < 0.05, ** *p* < 0.01.

In tauopathy, microglia transition from homeostatic to disease-associated states characterized by increased lysosomal activity and loss of homeostatic markers. Mounting evidence points to hyperactive microglial responses playing pivotal roles in driving tauopathy in AD patients as well as in tauopathy mouse models [1,2,3,5,37,38,39,40]. Therefore, we were next interested to explore how ZBP1 signaling impacts microglial activation in PS19 mice. To this end, we evaluated the staining of CD68 and P2RY12 on IBA1+ macrophages, which are canonical markers of microglial activation and homeostasis, respectively. Here, we found that the loss of ZBP1 in PS19 mice leads to a trend toward decreased coverage of the CNS macrophage marker IBA1 at 9 months of age (Figure 2A,B), which suggests a dampening of microglial responses in PZKO mice relative to PS19 littermate controls. In line with a potential diminishment of microgliosis in PZKO mice (Figure 2A,B), we also observed blunted expression of the phagolysosomal activation marker CD68 and markedly higher expression of the homeostatic microglial marker P2RY12 in PZKO mice (Figure 2A,C,D).

To gain a more comprehensive and unbiased assessment of how ZBP1 signaling affects microglial biology in tauopathy, we next performed single-nucleus RNA sequencing (snRNA-seq) on pooled hippocampi from 9-month-old PS19 and PZKO mice. Unsupervised clustering resolved 19 transcriptionally distinct populations visualized by UMAP (Figure S1A). Canonical marker identification (Figure S1B,C) identified major cell classes, including excitatory and inhibitory neurons (Ex and Inh), oligodendrocytes (OL), astrocytes (Astro), microglia (MG), oligodendrocyte progenitor cells (OPC), endothelial cells, pericytes (PC), ependymal cells (EP), vascular leptomeningeal cells (VLMC), and choroid plexus cells (CP) (Figure 2E). Cell-type proportion analysis showed similar representation of these major populations between PS19 and PZKO hippocampal samples (Figure S1D,E), indicating that ZBP1 deletion does not produce large-scale shifts in hippocampal cell composition.

Microglial nuclei (MG; cluster 3) enriched for *Csf1r*, *Cx3cr1*, and *Inpp5d* (Figure 2F), were next compared between genotypes. Differential expression analysis identified 1202 differentially expressed genes (DEGs) between PS19 and PZKO microglia (magenta = upregulated in PZKO; teal = downregulated in PZKO; Figure 2G). PZKO microglia showed coordinated downregulation of genes related to disease-associated microglia (DAM) and inflammatory signaling (e.g., *Apoe*, *Trem2*, *Tyrobp*, *C1qa*, *Itgax*), phagocytic and proteolytic activity (e.g., *Cd68*, *Lamp1*, *Cst7*, *Mpeg1*, *Ubb*), and antigen presentation (e.g., *Cd74*, *H2-D1*, *B2m*) (Figure 2G), suggesting that ZBP1 critically drives engagement of neurodegeneration-associated microglial activation programs in tauopathy. Notably, expression of the ZBP1 effectors *Irf3* and *Ripk1* was also reduced (Figure 2G), supporting a role for ZBP1 in amplifying tau-induced inflammatory signaling in microglia. Conversely, ZBP1-deficient microglia were found to express higher levels of TGF-β/SMAD3-associated signaling genes (e.g., *Smad3* and *Tgfbrap1*) relative to PS19 microglia (Figure 2G), consistent with a shift towards a more homeostatic microglial state in PZKO mice. Gene-set pathway enrichment of downregulated transcripts highlighted pathways related to microglial cell activation, synapse pruning, microglial phagocytosis, and complement activation (Figure 2H,I). Collectively, these data indicate that ZBP1 is a critical driver of microglial activation in the PS19 model of tauopathy.

### 3.3. Astrogliosis Is Reduced with the Deletion of ZBP1 in PS19 Mice

**Figure 3:**
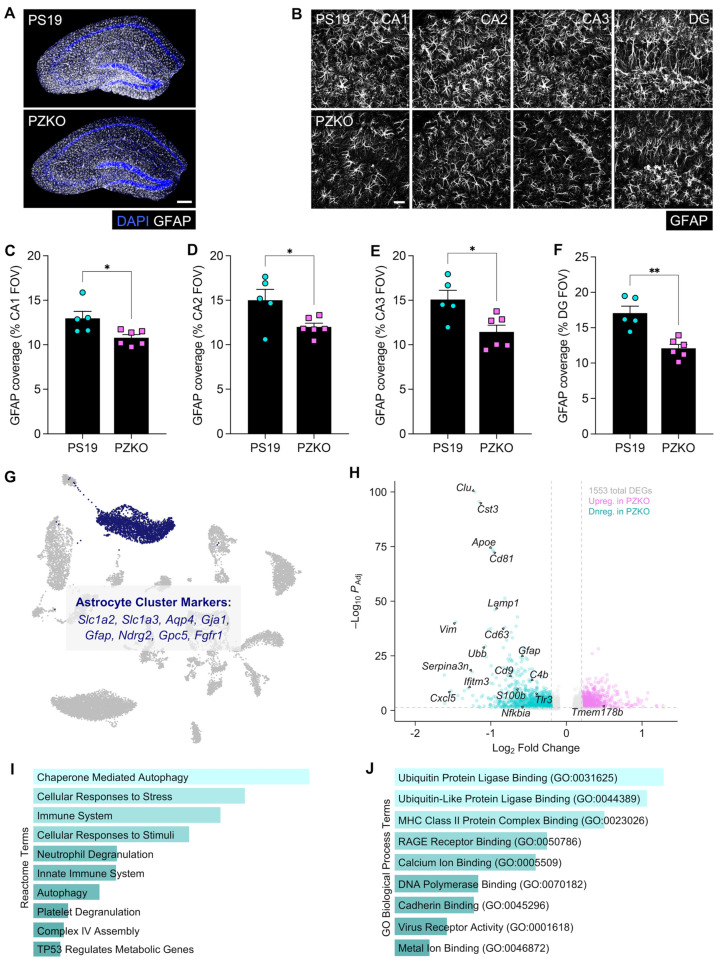
Astrogliosis is blunted with ZBP1 deficiency in PS19 mice. (**A**) Representative coronal hippocampal sections from 9-month-old PS19 and PZKO mice stained for DAPI (blue) and GFAP (white; scale bar = 200 µm). (**B**) Representative images of GFAP (white) in CA1, CA2, CA3, and DG (scale bar = 40 µm). (**C**–**F**) Quantification of GFAP^+^ area coverage per FOV in CA1, CA2, CA3, and DG. (**G**) snRNA-seq UMAP highlighting the astrocyte cluster with canonical markers. (**H**) Volcano plot depicting differential gene expression between PZKO astrocytes and PS19 astrocytes. (**I**) Reactome Pathway Database terms enriched in downregulated DEGs in PZKO astrocytes. Plot shows the top significantly enriched terms, ranked by ascending adjusted *p*-value. (**J**) GO Biological Process Database terms most significantly enriched in downregulated DEGs in PZKO astrocytes. Plot shows the top significantly enriched terms, ranked by ascending adjusted *p*-value. Statistical significance between experimental groups was calculated by linear mixed effects modeling. * *p* < 0.05, ** *p* < 0.01.

Astrocytes become reactive in tauopathy and contribute to inflammatory remodeling of the local neural microenvironment. To determine whether ZBP1 deletion alters astrocyte reactivity, we first evaluated GFAP^+^ coverage in the hippocampus of 9-month-old PS19 and PZKO mice by immunofluorescence staining. These imaging studies revealed significant reductions in GFAP^+^ coverage in PZKO mice across hippocampal subregions (Figure 3A–F) in contrast to the dense networks of reactive astrocytes in PS19 brains, indicating that ZBP1 loss dampens tau-associated astrocyte activation.

To gain a deeper understanding of how ZBP1 signaling impacts astrocyte biology, we next turned to our snRNA-seq dataset. To this end, astrocyte nuclei (AS; cluster 2) were enriched for canonical markers including *Aqp4*, Gja1, and *Gfap* (Figure 3G) and compared between genotypes. Differential expression analysis identified 1553 DEGs between PS19 and PZKO astrocytes (Figure 3H). PZKO astrocytes showed broad downregulation of genes associated with reactive and inflammatory states (Figure 3H). More specifically, we observed dampened expression of genes associated with astrocyte activation (*Gfap*, *Vim*, *Serpina3n*), metabolic distress and injury (e.g., Cst3, *Clu*, *Apoe*, *C4b*), stress responses (e.g., *S100b*, *Cxcl5*, *Ubb*), type I IFN signaling (e.g., *Ifitm3*, *Ifitm7*, *Tlr3*), and NF-κB signaling (e.g., *Nfkbia*) (Figure 3H). Consistent with these findings, gene-set pathway enrichment of downregulated transcripts highlighted pathways related to cellular stress responses, innate immune signaling, and autophagy (Figure 3I–J). Together, these imaging and transcriptional data indicate that ZBP1 deletion attenuates astrocyte reactivity and inflammatory responses in the hippocampus of PS19 mice.

### 3.4. ZBP1 Deletion Protects Against Neuronal Loss in Tauopathy

**Figure 4:**
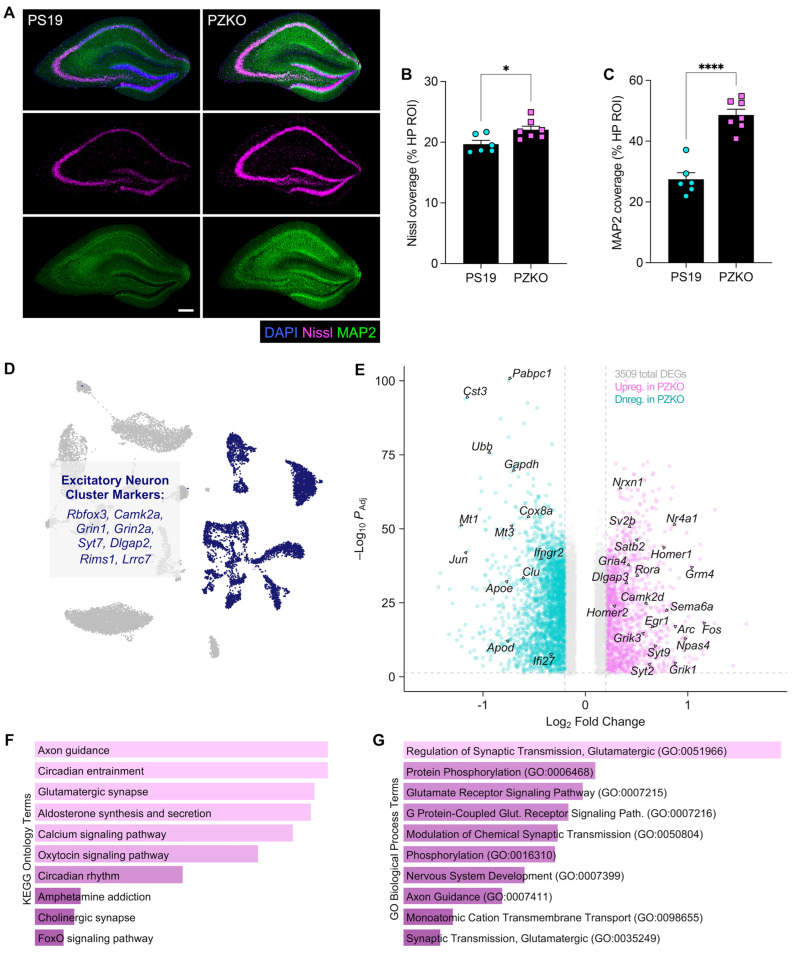
Loss of ZBP1 limits neuronal loss in tauopathy. (**A**) Representative coronal hippocampal sections from 9-month-old PS19 and PZKO mice stained for DAPI (blue), Nissl (magenta), and MAP2 (green; scale bar = 200 µm). (**B**) Quantification of Nissl^+^ area coverage per hippocampal ROI. (**C**) Quantification of MAP2^+^ area coverage per hippocampal ROI. (**D**) snRNA-seq UMAP highlighting the astrocyte cluster with canonical markers. (**E**) Volcano plot depicting differential gene expression between PZKO excitatory neurons and PS19 excitatory neurons. (**F**) KEGG Ontology Database terms most significantly enriched in upregulated DEGs in PZKO excitatory neurons. Plot shows the top significantly enriched terms, ranked by ascending adjusted *p*-value. (**G**) GO Biological Process Database terms most significantly enriched in upregulated DEGs in PZKO excitatory neurons. Plot shows the top significantly enriched terms, ranked by ascending adjusted *p*-value. Statistical significance between experimental groups was calculated by linear mixed effects modeling. * *p* < 0.05, **** *p* < 0.0001.

Intracellular tau aggregates disrupt the neuronal cytoskeleton, impair axonal transport and synaptic transmission, and ultimately drive the progressive neuronal loss that underlies cognitive decline. To determine how ZBP1 deletion affects neuronal health in tauopathy, we performed Nissl and MAP2 immunofluorescence staining to evaluate neuronal loss in PZKO mice and PS19 littermate controls at 9 months of age. In these studies, we observed increased coverage of both Nissl and MAP2 staining in PZKO hippocampal sections when compared to PS19 controls (Figure 4A–C), suggesting that ZBP1 deletion mitigates tau-associated neuronal degeneration.

We next leveraged our snRNA-seq dataset to further define how ZBP1 deletion affects gene expression signatures in excitatory neurons (Figure 4D), as dysfunction in this neuronal subset is known to be a major driver of disease in AD and tauopathies [41]. Differential expression analysis between PZKO and PS19 mice identified 3509 DEGs in this cluster (Figure 4E). PZKO excitatory neurons showed increased expression of glutamatergic receptor genes (e.g., *Gria4*, *Grik1*, *Grik3*, *Grm4*), activity-dependent plasticity genes (e.g., *Fos*, *Arc*, *Npas4*, *Egr1*, *Nr4a1*), and synaptic scaffolding/vesicle machinery (e.g., *Homer1/2*, *Dlgap3*, *Syt2*, *Syt9*) (Figure 4E). ZBP1-deficient excitatory neurons in PS19 mice were also found to upregulate genes linked to excitatory identity, wiring, and dendritic signaling (e.g., *Satb2*, *Nrxn1*, *Rora*, *Sv2b*, *Sema6a*, *Camk2d*) (Figure 4E). In contrast, PS19 excitatory neurons were relatively enriched for AD risk and injury/stress-associated genes (e.g., *Apoe*, *Clu*, *Cst3*, *Apod*, *Mt1*, *Mt3*, *Ubb*, *Jun*), housekeeping and metabolic genes (e.g., *Cox8a*, *Gapdh*, *Actb*), and IFN-linked genes (e.g., *Ifngr2*, *Ifi27*, *Pabpc1*) (Figure 4E). Gene-set pathway enrichment of upregulated transcripts in ZBP1-deficient neurons further highlighted pathways related to axon guidance and the regulation of glutamatergic synaptic transmission (Figure 4F,G). Together with increased Nissl and MAP2 coverage, these transcriptional changes indicate that ZBP1 deletion in PS19 mice protects against neurodegeneration and leads to increased expression of various neuronal health genes that are associated with synaptic transmission and plasticity.

## 4. Discussion

In this study, we found that genetic ablation of ZBP1 provides protection against tau pathology and neuronal loss in the PS19 mouse model of tauopathy. This protection against tau-mediated neurodegeneration in ZBP1-deficient mice was accompanied by dampened microglial activation and a transcriptional shift toward a more homeostatic phenotype. Moreover, we observed that ZBP1 deletion led to reduced coverage of reactive astrocytes, suggesting decreased astrogliosis. These findings support a model in which ZBP1 amplifies tau-induced inflammation and neurodegeneration in tauopathy, and highlight ZBP1-dependent signaling as a potential target to limit tau-driven neurodegenerative disease.

In recent years, there has been growing appreciation for the role of cytosolic nucleic acid-sensing pathways in neurodegenerative diseases [1,2,3,4,5]. These pathways bind DNA and RNA that accumulate in the cytosol from nuclear and mitochondrial sources, following exposure to replicative or oxidative stress [6,7,8]. Multiple studies have reported overactivated nucleic acid-sensing pathways in a variety of neurodegenerative diseases, including amyotrophic lateral sclerosis, Parkinson’s disease, and primary tauopathies [42]. For instance, dampening innate immune responses via cGAS-STING, an innate immune pathway that binds cytosolic double-stranded DNA, has been shown to alleviate pathological outcomes in animal models of amyloidosis, tauopathy, ALS, and Parkinson’s disease [2,10,11,12,42,43,44,45,46]. However, the contributions of other innate immune pathways have not been thoroughly explored in models of tauopathy.

ZBP1 is a recently identified interferon-stimulated sensor of Z-form nucleic acids (Z-NAs) that plays vital roles in the generation of protective immune responses to viral infection [18,19,20,21,22] and tumors [47,48,49,50]. Once activated, ZBP1 signals through RHIM-dependent interactions with RIPK1 and RIPK3 to promote MLKL-dependent necroptosis and caspase-8–mediated apoptosis. ZBP1 also amplifies inflammatory responses by enhancing NF-κB activation, promoting inflammasome assembly, and reinforcing type I IFN and interferon-stimulated gene (ISG) programs, thereby creating positive-feedback loops between nucleic acid sensing, inflammation, and cell death.

Emerging data suggest that Z-NAs can form in the absence of infection, and this has been shown to trigger ZBP1 activation and promote disease progression in a range of sterile disorders [23,24,25,26,27,28,29,30]. Interestingly, evidence from multiple independent groups has revealed that Z-NAs can be readily detected in post-mortem brain sections isolated from AD patients and further shows that Z-NA levels positively correlate with neurodegenerative disease severity [31,32,33]. Moreover, recent studies suggest that ZBP1 is centrally involved in promoting neuroinflammation and amyloid beta plaque deposition in the 5xFAD mouse model of AD-related amyloidosis [31]. These findings, coupled with those featured in this paper showing marked protection against tauopathy progression, position ZBP1 as an attractive therapeutic target to pursue in the treatment of AD, primary tauopathies, and the many other neurodegenerative disorders where genotoxic stress is thought to play a role.

## Data Availability

The original sequencing data presented in the study are openly available in the Gene Expression Omnibus (GEO) repository under accession number GSE325730.

## Supplementary Materials

The following supporting information can be downloaded at: https://www.mdpi.com/article/10.3390/cells15070591/s1, Figure S1: snRNA-seq of pooled hippocampus, clustering, and canonical marker-based cell-type identification. (**A**) UMAP plot depicting numbered and annotated clusters identified by single-nucleus RNA sequencing (snRNA-seq) of hippocampal tissue pooled from PS19 and PZKO mice at 9 months of age. (**B**) Dot plot depicting the expression of canonical marker genes (‘Features’) in individual clusters (‘Identity’), colored by average expression (purple = high; cyan = low) and scaled by percent expressed. (**C**) Graph depicting the percentages of each major identified cell type out of total combined nuclei across groups. (**D**) Graph depicting the fraction of nuclei of each cell type, split by genotype. (**E**) Heat map depicting the top 4 enriched markers for each cluster relative to all others and across groups. Column color labels correspond to the color key for clusters in (**A**). Normalized expression is depicted as a color scale (black = high; light grey = low).
