# Supplementary material for: The Genotoxic Stress Sensor ZBP1 Drives Tau Pathology
Source: Cells. 2026 Mar 26;15(7):591. doi: 10.3390/cells15070591 (PMC13072345; doi:10.3390/cells15070591)
Supplement: Supplementary file 1 [file cells-15-00591-s001.zip › cells-4169467-supplementary.pdf]

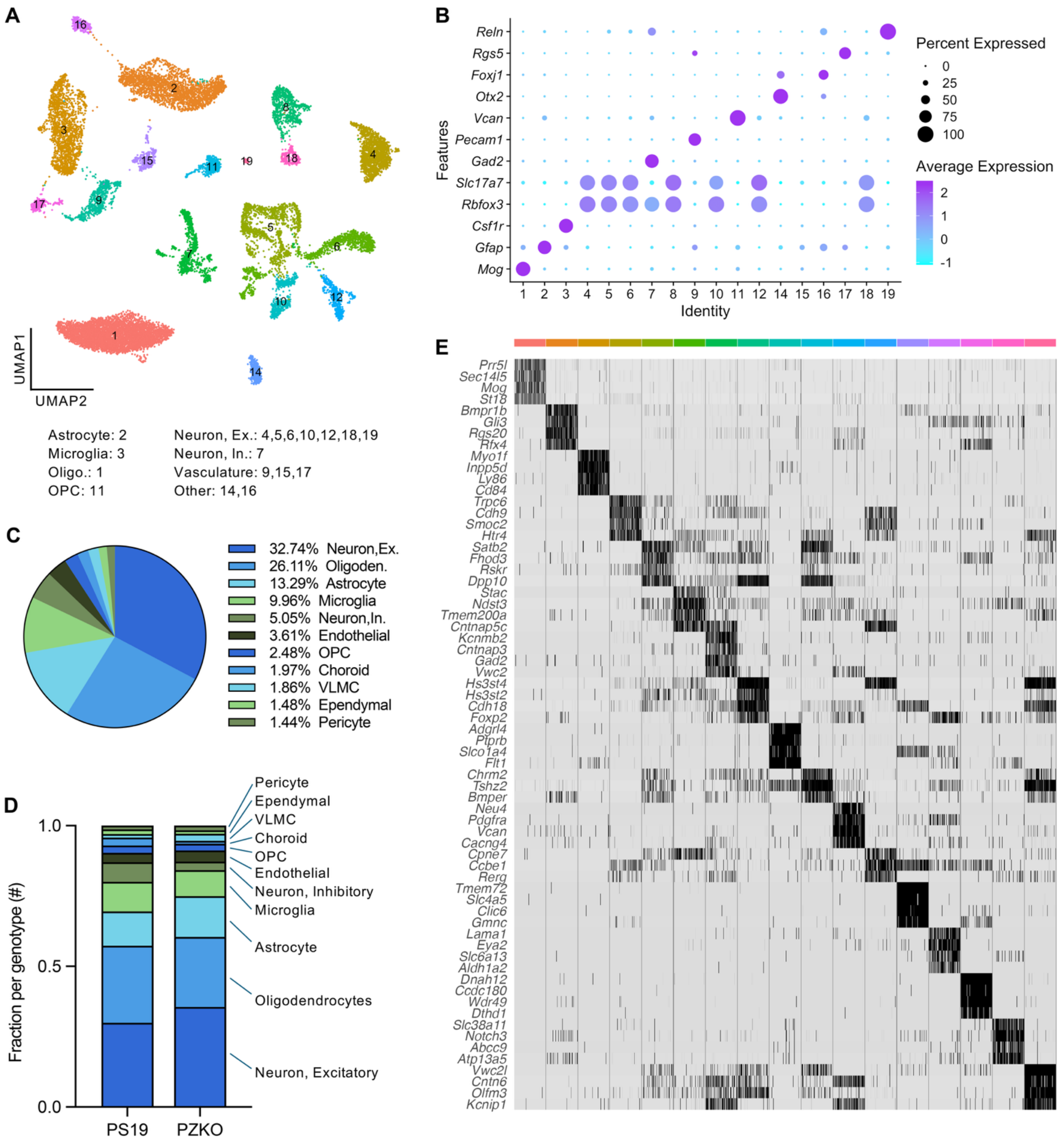

Figure S1. snRNA-seq of pooled hippocampus, clustering, and canonical marker-based cell-type identification. (**A**) UMAP plot depicting numbered and annotated clusters identified by single-nucleus RNA sequencing (snRNA-seq) of hippocampal tissue pooled from PS19 and PZKO mice at 9 months of age. (**B**) Dot plot depicting the expression of canonical marker genes ('Features') in individual clusters ('Identity'), colored by average expression (purple = high; cyan = low) and scaled by percent expressed. (**C**) Graph depicting the percentages of each major identified cell type out of total combined nuclei across groups. (**D**) Graph depicting the fraction of nuclei of each cell type, split by genotype. (**E**) Heat map depicting the top 4 enriched markers for each cluster relative to all others and across groups. Column color labels correspond to the color key for clusters in (**A**). Normalized expression is depicted as a color scale (black = high; light grey = low).
